# A closer look at lithium-ion batteries in E-waste and the potential for a universal hydrometallurgical recycling process

**DOI:** 10.1038/s41598-024-67507-7

**Published:** 2024-07-19

**Authors:** Johannes J. M. M. van de Ven, Yongxiang Yang, Shoshan T. Abrahami

**Affiliations:** Department of Materials Science and Engineering, Delft University of Technology, Mekelweg 2, 2628 CD Delft, The Netherlands

**Keywords:** Chemical engineering, Inorganic chemistry

## Abstract

The demand for lithium-ion batteries (LiBs) is rising, resulting in a growing need to recycle the critical raw materials (CRMs) which they contain. Typically, all spent LiBs from consumer electronics end up in a single waste stream that is processed to produce black mass (BM) for further recovery. It is desired to design a recycling process that can deal with a mixture of LiBs. Hence, this study investigates the structure and composition of battery modules in common appliances such as laptops, power banks, smart watches, wireless earphones and mobile phones. The battery cells in the module were disassembled into cell casing, cathode, anode and separator. Then, the cathode active materials (CAMs) were characterized in detail with XRD-, SEM-, EDX- and ICP-OES-analysis. No direct link was found between the chemistry of the active materials (NMC, LCO, LMO, LFP etc.) and the application. Various BM samples were submitted to a leaching procedure (2 M H_2_SO_4_, 50 °C, 2 h, 60 g BM/L) with varying concentration (0–4 vol%) of H_2_O_2_ to study the influence of their chemical composition on the dissolution of Li, Ni, Mn and Co. Only a part of the BMs dissolved completely at 4 vol% H_2_O_2_, which was attributed to the oxidation state of the transition metals (TMs). Exact determination of H_2_O_2_ consumption by redox titration confirmed this hypothesis.

## Introduction

In the current society, batteries are widely used for storage of energy; from green technology applications, such as electric vehicles (EVs) and storage units for intermittent renewable energy sources (such as solar and wind), to consumer electronics such as mobile phones and laptops^1^. Rechargeable lithium-ion batteries (LiBs) are the most prevalent type of batteries in such applications, with their demand growing, while the supply of the necessary materials is under pressure^2,3^. The rapid pace of technological advancements and consumer preferences for frequent device upgrades have contributed to a significant increase in e-waste generation^4^. A substantial portion of this e-waste nowadays contains LiBs, which pose severe safety and environmental concerns when not properly managed^5–7^. Although batteries in consumer electronics are a relatively small fraction of the global market, all batteries are subject to European Union (EU) regulations and need to be (manually) removed from their devices for subsequent treatment before further e-waste processing and recycling^8–10^.

LiBs contain many elements that are currently listed as critical raw materials (CRMs) according to the EU, such as cobalt, manganese and lithium^11–14^. Recycling can partly relieve pressure on primary resources, thereby reducing the reliance on import^14,15^. Although this already happens to some extent, industrial recycling processes that are in place focus on the most valuable elements, such as cobalt, nickel and copper^12,16,17^. Therefore, there is a need for recycling processes that aim to recover all elements. Most studies focussed on optimizing the hydrometallurgical recycling of a single black mass (BM, a mixture of LiB cathode materials and impurities) composition, obtained from dismantling batteries by hand or through simulated crushing processes^18–26^. For example, H_2_SO_4_ (1–3 M) is a widely used for the leaching of cathode materials^19,27^. However, complete dissolution is often not reached without high temperatures (up to 95 °C) and/or long reaction times (up to 6 h). Also, additional reagents such as H_2_O_2_ are used to improve leaching for Li, Mn, Co and Ni at milder conditions^20,22,28^. For example, Sattar et al.^22^ used a solution of 2 M H_2_SO_4_ with 4 vol% H_2_O_2_, resulting in a leaching efficiency ≥ 98% for Li, Co, Ni and Mn (50 °C, 2 h, S/L =  ± 60 g/L). They used one mixture of heat treated CAMs from manually dismantled electronics as BM. In another study, He et al.^28^ acquired ≥ 99.7% leaching efficiency for these four elements with only 1 M H_2_SO_4_ and 1 vol% H_2_O_2_ (40 °C, 1 h, S/L = 40 g/L), using pristine NMC 111 cathode powder. HCl solutions of up to 4 M are also used and result in high (≥ 99%) leaching efficiencies for Li, Ni, Mn and Co^18,25,26,29^. These processes required higher temperatures (80 °C) but differs in the required leaching time (1–2 h), as well as in the reported S/L ratio (20–100 g/L). Other studies describe leaching processes with HNO_3_^30,31^ and H_3_PO_4_^21,32^, or organic acids such as oxalic acid^33,34^, citric acid^24,35^ and acetic acid^36,37^, sometimes with additional reagents such as H_2_O_2_. Since the feed material differs per study; ranging from pristine materials to (a mixture of) cathode materials from used batteries, the influence of feed chemistry on leaching was not directly studied. Therefore, the reality of contemporary industrial e-waste and battery sorting and the following (mechanical) processing capabilities are not taken into account^38^. For example, it is not possible to distinguish the specific chemistry of a Li-ion battery by simple visual inspection of its casing (though new regulations regarding battery passport are predicted to change this in the next decade)^39,40^.

As a consequence, industrial scale BM preparation processes will inevitably process a mixture of the various types of LIBs available on the market. Also, despite the available technologies for mechanical separation, additional materials originating from different components of the module (e.g. metallic materials in the casing and battery management system) will likely introduce extra contaminates to the BM. This drastically increases the heterogeneity of input material to downstream recycling processes and may causes limitations in the achieved purity levels. To explore the potential of a flexible and universal hydrometallurgical recycling process, this paper reports on the first step in achieving such an aim by leaching various types of different BM compositions. Therefore, the present study begins with characterizing the structure of battery modules and cell chemistry in various small electronic appliances such as laptops, mobile phones, power banks, smart watches, and wireless earphones. Next, the influence of composition on the recycling process of the main present metals (Li, Co, Mn and Ni) is investigated. Finally, the potential of establishing a more flexible and universal hydrometallurgical processing route is discussed.

## Experimental

### Battery retrieval and discharging

To get an insight in the composition of battery waste from consumer electronics, a variety of end-of-life (EoL) devices were disassembled. These were provided by a local collector of batteries (Van Peperzeel B.V.). Removal of the batteries from their respective devices was rather straight forward. The devices could either be screwed, pried or in some cases cut open with a Dremel. Then, a battery module (cell(s) with casing, Battery Management System (BMS) etc.) was removed from a device. These were disassembled and their components were weighed, after which the weight percentages for all components were calculated. The retrieved battery cells were then discharged by submerging them in an aqueous K_2_CO_3_ (≥ 99.0%, Sigma-Aldrich) solution of 10 wt% for (at least) 24 h^41^. Next, the cells were wiped off and dried in an oven at 50 °C for 8 h. The voltage was measured before and after treatment to verify a successful discharge.

### Cell dissmantling

Two cell types could be distinguished: cylindrical (18650) and pouch cells. The cylindrical cells have a nickel-plated steel casing and were opened by cutting the top and bottom of the battery with a pipe cutter, after which a longitudinal cut was made (Fig. S1, Supplementary information). After this, the steel casing could be removed, revealing the cathode, separator, and anode. The pouch cells have plastic casings, which can be easily cut open with a knife. For both types of cells, the electrodes and separator were uncurled, after which they could be separated. All parts were weighed and compared to the initial cell weight to account for any losses during the dismantling process. The mass percentages for all components were also calculated. The electrolyte is not retrieved separately and mostly evaporates after opening the cell. It is accounted for in the “loss” category.

### Preparation of black mass (BM)

After separation of the cathode from all other cell parts, it was cut into pieces of roughly 1.5 × 1.5 cm. These pieces were submerged in N-methyl-2-pyrrolidone (NMP) (99%, Thermo Scientific), which dissolved the binder. This process was done at 75 °C with ultrasonification (Emag Emmi-40HC ultrasound bath). Depending on the battery, this took between 1 and 3 h. After NMP treatment, the solution was left to cool down and the aluminium foil pieces were filtered out. The NMP with cathode material in suspension was left to settle for at least 24 h, after which it was decanted, leaving a dense slurry of NMP and BM (CAMs with impurities). Both the aluminium foil and BM were dried at 60 °C until no solvent was left. They were weighed and mass percentages compared to the total cathode weight were calculated. Around 65–80 wt% of the cathode was retrieved as BM. The rest consists of the aluminium foil, as well as the removed binder and cathode material in suspension of NMP which could not be fully filtrated due to the small particle size. The retrieved BM samples were subjected to various characterization techniques, as described in Section 2.4. In addition, Hanwa Europe B. V. provided an industrially pre-treated (mechanically processes and pyrolyzed) BM. Important to note here is that this method to retrieve BM differs substantially from the liberation methods used in industry^42^. Industrial pre-treatment results in BM with many impurities from other parts of the battery (cells), as shown later in this study. For research purposes the aim is to minimise the amount of impurities in the BM, which is why a delicate liberation method is chosen. Pristine NMC 532 was bought from Nanographi.

### Characterization techniques

The different phases in the black mass were analysed through X-ray diffraction (XRD) analysis with a Bruker D8 Advance diffractometer with Bragg–Brentano geometry and a Lynxeye position sensitive detector, using Cu Kα radiation. The 2Ɵ range was 10°–110°, with a step size of 0.04° and a counting time of 2s per step. Bruker software DiffracSuite. EVA vs. 6.0 was used for data analysis.

The morphologies and individual grain compositions of various black mass samples were investigated using a scanning electron microscope in combination with energy dispersive X-ray analysis (SEM–EDX). For this, a Jeol JSM-IT100 was used. A small amount of black mass was applied on a piece of carbon tape and placed on the sample holder.

Before analysing the black mass with ICP-OES, it was dissolved in aqua regia to ensure complete dissolution. Each black mass type was analysed three times to account for heterogeneity. 100 mL of aqua regia was prepared by combining 25 mL of HNO_3_ solution (65%, VWR chemicals) with 75 mL HCl solution (37%, Merck). This was added to a triple necked round bottom flask with reflux cooler. In this, 1 g of black mass was dissolved at 70 °C for 5 h, while stirring at 500 rpm. This entire solution was filtered through a Whatman 595 ½ folded filter paper and diluted to 1 L, using a volumetric flask. Samples from this solution, as well as the diluted PLS samples, were applied for ICP-OES analysis with a Spectro Arcos-EOP-device, with Modified Lichte nebulizer and mini cyclon spray chamber. In order to verify complete dissolution of all metals, the residue was analysed with SEM in combination with EDX-analysis. In order to avoid any matrix effects during ICP-OES analysis, both the samples from digestion and leaching were diluted a second time with a 3 wt% HNO_3_ (65%, VWR chemicals) solution, with a dilution factor of 20.

### Leaching

The lixiviant was prepared by adding concentrated sulfuric acid (95–97%, Sigma-Aldrich) to Milli-Q water in a glass vial of 30 mL. In some cases, H_2_O_2_ solution (30%, Sigma-Aldrich) was added as well. To account for bubble formation, the maximum total amount of lixiviant was kept at 16 mL and a small hole was made on the top of the screw cap. A magnetic stir bar was added, after which the black mass was weighed and added to the lixiviant. The glass vials containing the leaching system were then placed in an aluminium heating block on a stirring plate with a thermocouple, at 50 °C while stirring at 400 rpm. After 2 h maximum dissolution is reached^22^. The leaching system was removed and a sample of the pregnant leach solution (PLS) was taken. This was filtered with a syringe filter (Chromafil Xtra PFTE-45/25), after which 0.2 mL PLS was diluted to 10 mL and submitted to ICP-OES analysis. To ensure a minimal error due to water evaporation, the initial and remaining volume were compared. Also, the residue was analysed to ensure a correct mass balance. Each leaching experiment was done in triplicate.

To evaluate the leaching performance, the leaching efficiency (η_L_) of each experiment was calculated according to Eq. (1). In this formula, m^x^_f_ represents the initial mass of element x in the feed and m^x^_PLS_ represents the mass of element x present in the PLS after leaching.

1 $${\eta }_{L}= \frac{{m}_{PLS}^{x}}{{m}_{f}^{x}}*100\%$$

### KMnO_4_ titration

To determine and compare the necessary amount of H_2_O_2_ for full dissolution of the black mass samples, a leaching experiment was performed with an excess H_2_O_2_. The same leaching procedure was followed as described earlier, with 10 vol% of H_2_O_2_ solution. After leaching, 0.2 mL of the PLS was diluted and submitted to ICP-OES analysis. The remaining solution was diluted and directly titrated with a 0.3 M KMnO_4_ (EMSURE, ACS grade) solution to determine the remaining amount of H_2_O_2_^43,44^. This was used to calculate the amount of H_2_O_2_ that was used in the dissolution.

## Results and discussion

### Battery modules composition

**Figure 1:**
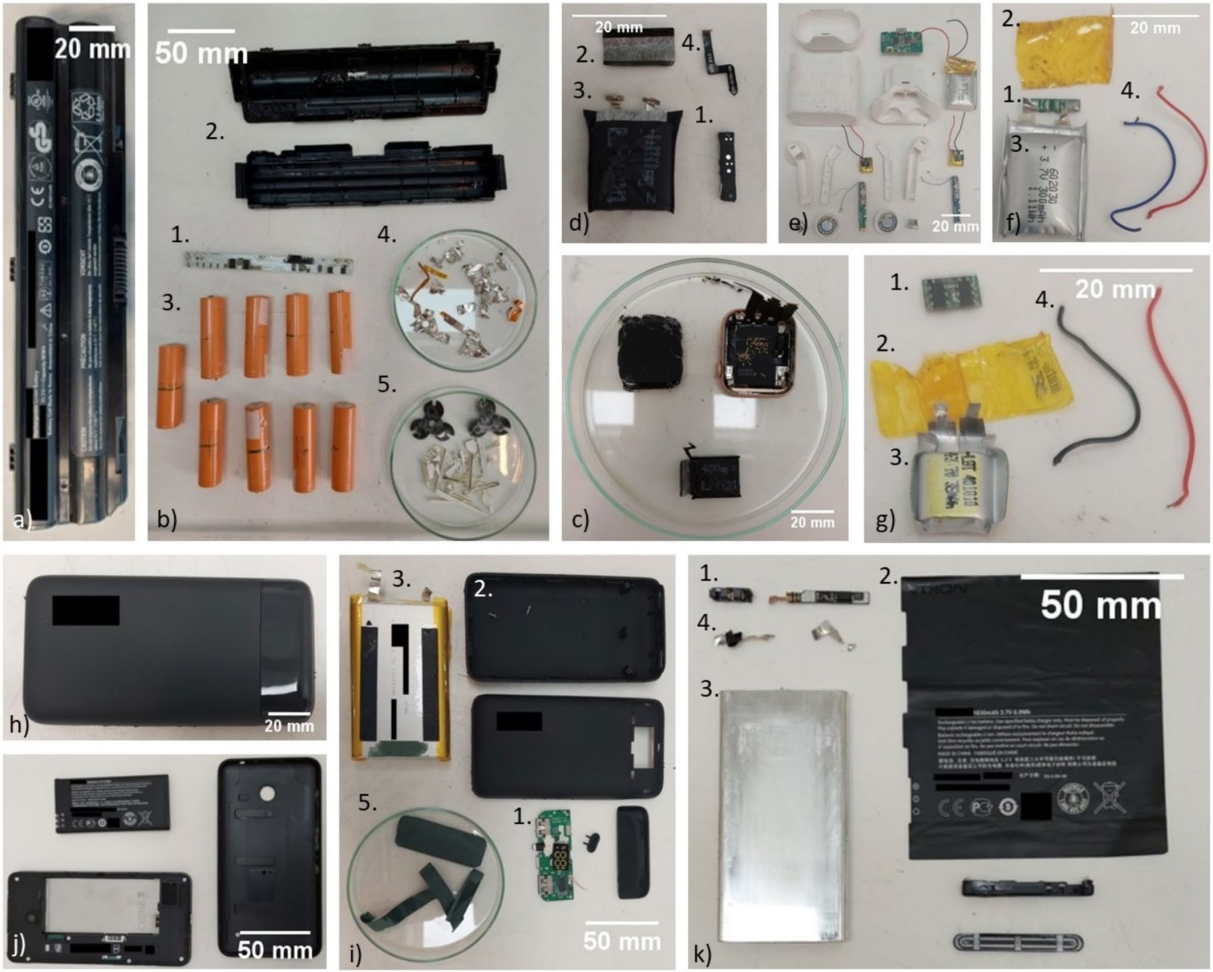
Battery modules (some of which with device) before and after disassembly. Represented are a (disassembled) laptop battery module (**a**, **b**), smart watch (**c**, **d**), set of wireless earphones (**e**, **f**, **g**), powerbank (**h**, **i**) and mobile phone module (**j**, **k**). For all sub images, the numbers represent the same component: 1. BMS, 2. Battery casing, 3. Battery cells, 4. Wiring, 5. Glue and separating components.

**Figure 2:**
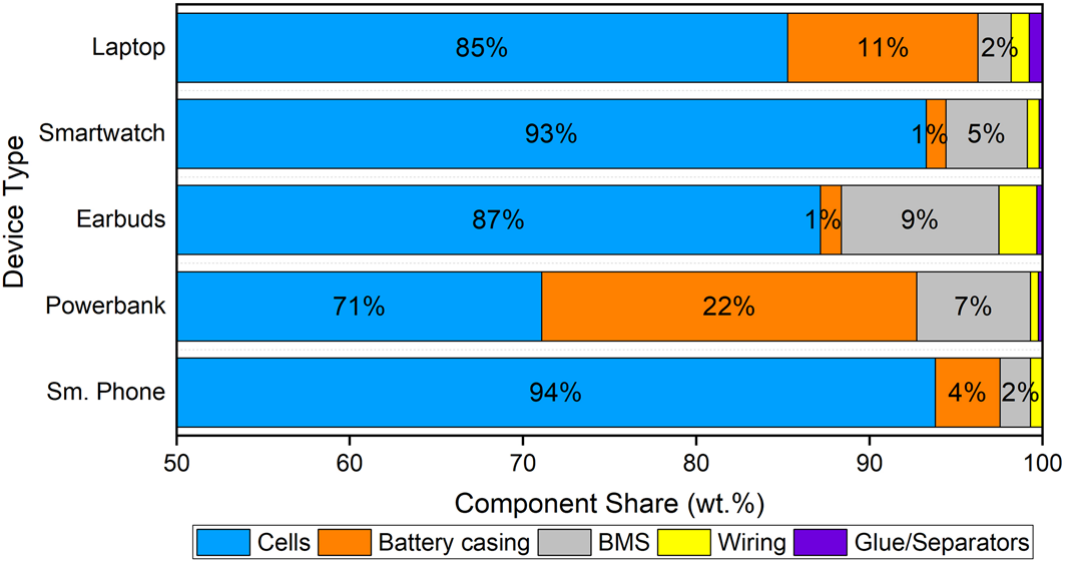
Proportional composition of battery modules from laptops, smartwatches, earbuds, powerbanks and (smart)phones (sm. phone). Note that the x-axis starts at 50 wt%.

Some very prevalent devices with LiBs are laptops, smartwatches, wireless earbuds, powerbanks and mobile (smart) phones^1^. From each of these applications, multiple battery modules have been disassembled. In Fig. 1, one example of a battery module (sometimes with respective device) can be seen for each mentioned application. The battery module is made of five major components: the battery cell(s), battery casing, the battery management system (BMS), wiring and separators or glues. The battery cells contain the active materials and are used for the storage of electrical energy. The wiring connects multiple cells to the BMS, which in turn ensures safe charge and discharge of the battery cells. The separators are used to avoid short circuiting by direct contact of the cells, and the glue keeps the cells in place during use. Lastly, the casing encapsulates and protects all other components. After disassembly, the average relative weights of all components from the battery modules were calculated. These are compared in Fig. 2 for the main five components.

The laptops (3 devices) researched in this study all included a rather easily removeable module, one of which is shown in Fig. 1a. Figure 1b shows this same battery module after disassembly. For this application the cells, either 18650 type as seen in the picture or pouch type, take up most of the weight (85 wt%). This is followed by the battery casing with 11 wt%. The BMS (1.5 wt%), glue and separators (1.0 wt%) and wiring (0.8 wt%) are only minor components.

The disassembled smartwatches (4 devices) use a smaller battery module with one cell (Fig. 1c). These modules consist of a pouch type cell (93 wt%), to which a BMS (5 wt%) is attached. The battery casing consists of a plastic foil (1.2 wt%) wrapped over the BMS. Hard plastic casings, such as seen in the laptop battery modules, are not present. The wiring and glue or separators have a very low relative weight of 0.7 and 0.2 wt% respectively. An example of the disassembled module is shown in Fig. 1d.

All disassembled earbud sets (4 devices) (Fig. 1e) contain three battery modules: one large module (Fig. 1f) and two smaller modules (Fig. 1g). These mostly consist of a cell (87 wt%) to which a BMS is attached (9 wt%). It must be noted that the BMSs shown in Fig. 1f and g are not present in all modules that were retrieved. In two units, the cells are directly attached to the circuitry in the earplugs or the casing, avoiding the need for an extra BMS. The battery casing consists, similarly to the modules present in smartwatches, of a foil wrapped around the module (1.2 wt%). The wiring and glue or separators contribute 2.2 and 0.3 wt% respectively.

An example of a powerbank is shown in Fig. 1h. Figure 1i shows this powerbank after disassembly. The disassembled powerbanks (5 devices) contain one to three cells, either 18650 or pouch type, which take up 71 wt% on average. The casing and BMS take up 22 and 6.6 wt% respectively. The wiring and glue or separators are only minor components, taking up 0.5 and 0.3 wt% respectively.

The array of (smart) phones (10 devices) that were disassembled contain two types of battery modules, all with one single pouch type cell. One module type is similar to the modules found in the smartwatches and earbuds, albeit larger. The second type is shown in Fig. 1j and is easily removeable from the device. These were generally found in the older (smart) phones (2008–2015), but it also depended on the brand. The module after disassembly can be seen in Fig. 1k. The cell (94 wt%) is similar in shape to the pouch cells seen in Fig. 1i, except for the connectors. Instead of a broad wire, flat connecting points are used on which the BMS (1.7 wt%) is directly applied. Some modules such as the one shown make use of wiring (0.7 wt%) to attach the BMS to (one of) the connecting points. The module casing consists of a sticker wrapped around the cell, in combination with plastic parts to cap off the top and bottom of the module (3.7 wt%). Glue and separators are used very scarcely (< 0.1 wt%).

When comparing the different applications with each other, major variations can be seen. The powerbanks and laptop battery modules have multiple cells which need to be kept in place, connected with wiring and isolated by rubber pads to avoid short circuiting. Therefore, the relative weight of the cells is lower (71–85 wt%) than for applications with just one cell per module (87–94 wt%). The smaller modules consist of one cell (87–94 wt%) to which the other parts are attached. These modules are generally not easily removed from the device since they are kept in place by a glue. In this manner, the device itself also acts as protective cover. Therefore, the need for casing, internal wiring and separators is limited. This results in a large weight contribution of the battery casing in the applications with multiple cells (11–22 wt%) compared to applications with one cell per module (1–4 wt%). Divergent are the easily removable batteries from (older) mobile phones, as seen in Fig. 1j, of which the casing contains hard plastic parts to protect the BMS during removal and placement. This results in the higher casing contribution for the mobile phones. The BMS is relatively large for smaller modules, resulting in a high weight contribution for the earbuds (9.1 wt%) and smartwatches (4.7 wt%). The BMS contribution is lower for the larger modules from smartphones (1.7 wt%) and laptops (1.9 wt%). This does not hold for the powerbanks, since the BMS often also harbours a screen and multiple charging ports resulting in a higher relative weight. The two other components (wiring, glue and separators) are similar for all categories and mostly depend on the size of the module. It must be noted that the limited number of samples in this study provides a good illustration of the typical components in consumer e-waste stream containing LIBs, but is not exhaustive and further variation can be expected.

### Battery cells composition

**Figure 3:**
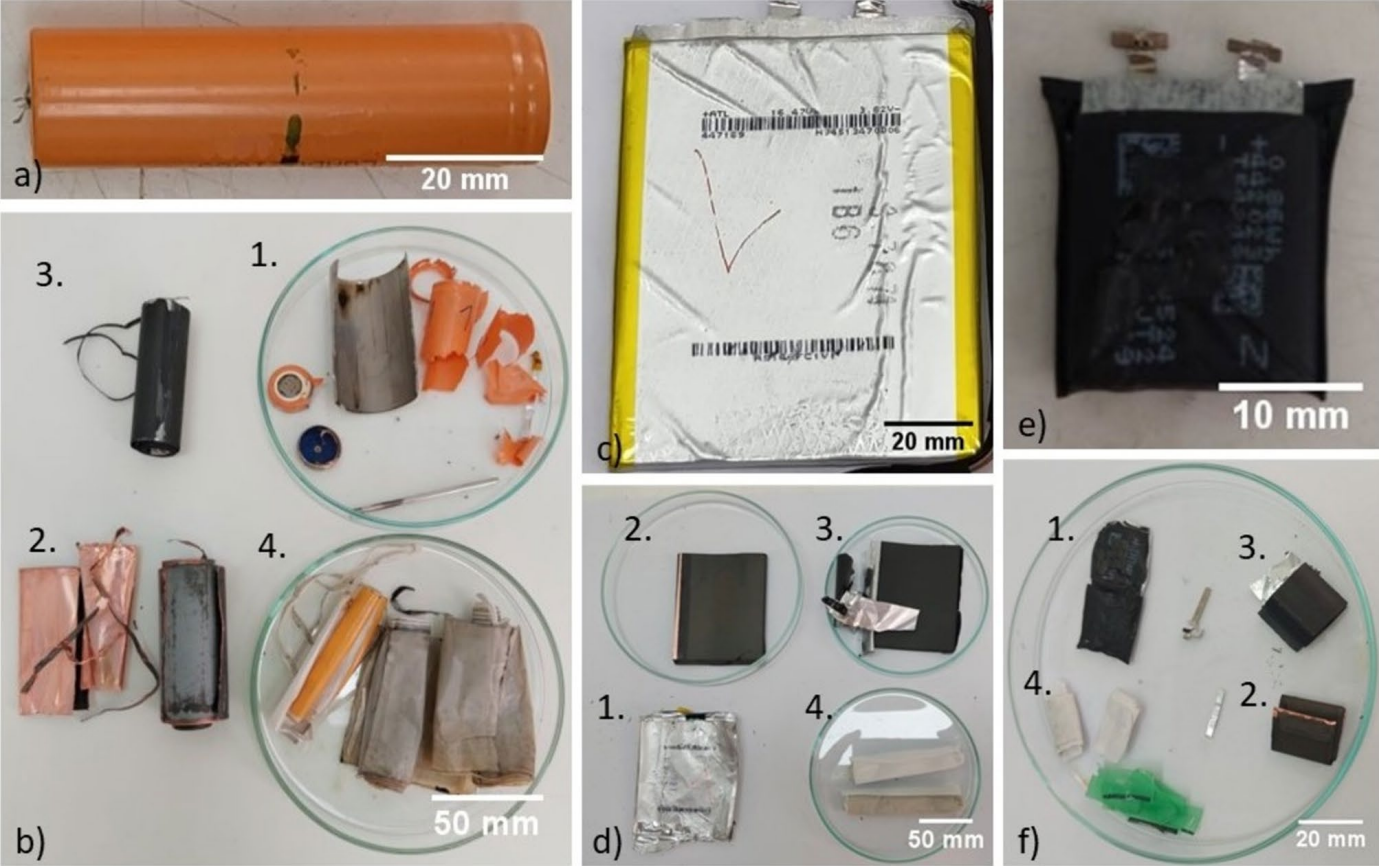
18650 type (**a**, **b**) and two pouch type (**c**–**f**) battery cells before and after disassembly. For all sub images, the numbers represent the same component. 1. Cell casing, 2. Anode, 3. Cathode, 4. Separator.

**Figure 4:**
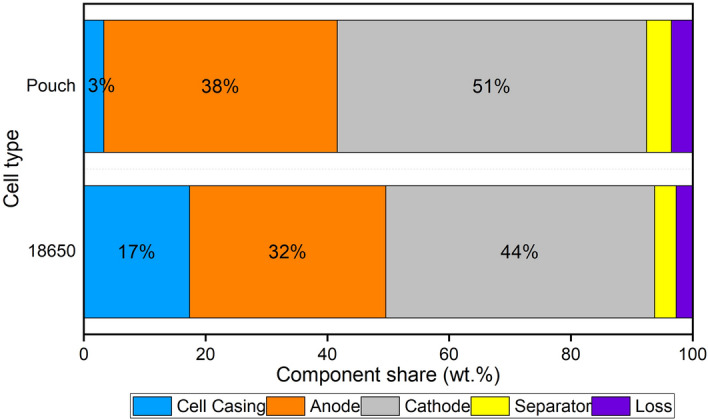
Average proportional composition of pouch and 18650 type cells. Subtraction of the component weights from the initial cell weight results in the loss category. It includes evaporated electrolyte and potential loss of (active) materials during cell opening.

After removing the cells from the battery modules, they were placed in the salt electrolyte for discharging before disassembly. Three typical examples can be seen in Fig. 3, before and after disassembly. The two most popular types of cells, cylindrical (typically 18650) and pouch cells, are shown. Although battery cells vary in size and shape, their basic structure is identical. Generally, a battery cell consists of the cell casing, an anode (graphite (C) on copper foil), a cathode (lithium metal oxide on aluminium foil) and a polymer separator with a supporting electrolyte in which lithium ions move^1^. The electrode foils (Cu, Al) are present to transfer electrons from and to the electrode materials via an external circuit. All components are indicated by the numbers 1–4 in Fig. 3. The average proportional composition of both cell types is compared in Fig. 4.

Figures 3a and b show a 18650 battery cell before and after disassembly, respectively. In these cells, the cathode is most prevalent (44 wt%), followed by the anode (32 wt%) and cell casing (17 wt%). The separator and loss have a low contribution of 3.6 and 2.7 wt%, respectively. Figure 3c and e both show pouch type cells. Figure 3d and f show these same cells after opening. Within these cells, the cathode has again the highest component share (51 wt%), followed by the anode (38 wt%). The cell casing, separator and loss all have a low contribution, being 3.3, 4.1 and 3.5 wt%, respectively.

When comparing the two cell types, the main difference is found in the contribution of the cell casing. This is much larger for the 18650 cells, which have a steel casing, compared to the pouch cells, with a plastic one. When the cell casing is excluded, the relative share of the other components is the same for both cell types. The composition of the 18650 cells found here is in good agreement with earlier studies when considering the contribution of the cell casing and separator^41,45^. Differences are found when comparing the anode and cathode, as well as the electrolyte. The latter is not considered in our study, since it is impregnated on the separator, cathode and anode. Also, a part of it evaporates after opening the cells (this is incorporated in the “loss” category). The electrolyte is therefore not selectively removed and weighed. This results in the anode and cathode categories being larger since they include some remaining electrolyte. Also, the amount of cathode or anode material that is attached to the collector foil can vary amongst different manufacturers, resulting in a different component share^46^.

### Black mass composition and morphology

**Figure 5:**
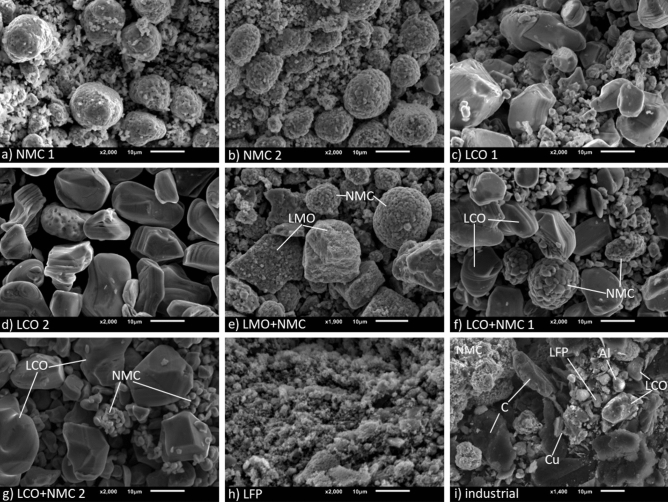
SEM images of BM retrieved manually from batteries (**a**–**h**) and industry (**i**). Individual grains were characterised by SEM–EDX.

To study the variety in composition and morphology of BM from Li-ion battery waste streams in consumer electronics, samples from the previously disassembled cells were characterized. The SEM results can be seen in Fig. 5. The explanation of these results, as well as the prevalent phases according to XRD, are presented in Table S1. Their elemental compositions according to ICP-OES are depicted in Table 1.

**Table 1:** Elemental composition (wt%) according to ICP-OES analysis of various manually acquired BM types, and one industrial sample.

Sample name	Li	Co	Ni	Mn	Al	Fe	Cu	PO_4_	Other
NMC1	5.6	12.2	28.7	17.2	0.4	0.1	/	/	35.6
NMC2	5.4	9.5	34.8	18.8	0.2	0.3	/	/	31.0
LCO 1	6.6	57.5	0.0	0.0	3.5	0.0	/	/	32.4
LCO 2	6.5	58.9	1.5	0.0	0.1	0.0	/	/	32.9
LMO + NMC	4.4	4.6	11.6	41.4	2.5	1.2	/	/	34.2
LCO + NMC1	7.4	53.8	6.6	3.1	0.1	0.0	/	/	29.2
LCO + NMC2	6.7	53.3	6.3	3.4	1.0	0.0	/	/	29.1
LFP	4.1	0.0	0.0	0.0	0.3	32.6	/	52.2	10.8
Industrial	4.2	18.1	12.3	4.7	2.8	0.4	2.1	/	55.4

“Other” category includes graphite, leftover binder and oxygen.

The first two samples, which are shown in Fig. 5a and b and labelled NMC 1 and NMC 2 respectively, both consist of a mixed lithium metal (nickel, cobalt and manganese) oxide. They are present as small particles (1–4 µm), as well as larger coagulates of these particles (8–20 µm). XRD-analysis confirms the presence of a mixed lithium metal oxide in both NMC 1 and NMC 2. ICP-OES-analysis (Table 1) shows that Ni is the most prevalent element in both samples (29–35 wt%), followed by Mn (17–18 wt%) and Co (10–12 wt%). The samples consist of around 5.5 wt% Li. Al is present as impurity in both samples (0.2–0.4 wt%). 31–36 wt% is taken up by other compounds or elements, such as graphite, binder and O.

The samples shown in Fig. 5c and d consist of an oxide of Li and Co, labelled as LCO 1 and LCO 2, respectively. The grain size ranges from 2 to 40 µm in LCO 1, and from 10 to 30 µm in LCO 2. According to XRD-analysis, both samples consist of LiCoO_2_, however LCO 1 also contains CoO_2_. ICP-OES-analysis shows that cobalt is the most prevalent element in both BMs (58–59 wt%), followed by Li (6.5–6.6 wt%). LCO 1 contains a small amount of Al as impurity (3.5 wt%), while LCO 2 contains some Ni (1.5 wt%). Both samples also contain 32–33 wt% of other compounds or elements.

Figure 5e, f and g show samples that consist of blended cathode material, called LMO + NMC, LCO + NMC 1 and LCO + NMC 2 respectively. These blended cathode materials are a more recent development in LiB technology, and will therefore be increasingly expected in e-waste in the coming decades^47,48^. Cathode blending is done to complement positive aspects of certain cathode chemistries, while also mitigating their drawbacks^48^. XRD-analysis indicates that the first sample consists of LiMn_2_O_4_ + Li_1.2_Mn_0.6_Ni_0.2_O_2,_ whereas the other two samples consist of LiCoO_2_ and LiNi_x_Mn_y_Co_z_O_2_. According to ICP-OES-analysis, LMO + NMC mostly consists of Mn (41 wt%), followed by Ni (12 wt%), Co (4.6 wt%) and Li (4.4 wt%). Al is present as impurity (2.5 wt%). Both LCO + NMC samples mostly consist of Co (54–53 wt%). Ni (6.6–6.3 wt%) and Mn (3.1–3.4 wt%) are present in lower amounts. Both also contain a small amount of Al (0.1–1 wt%). Other compounds take up 34.2, 29.2 and 29.1 wt% respectively.

Figure 5h shows BM that is retrieved from lithium iron phosphate (LFP) battery cells. This type of cathode material is increasingly used due to its higher stability during charge and discharge and its lower cost^49,50^. However, its chemical properties also result in a lower capacity^49,50^. The grains look more like flakes and range from 1 to 15 µm in size. According to XRD-analysis, this sample consists of LiFePO_4_, FePO_4_ and graphite. ICP-OES-analysis shows that of the LFP black mass, 33 wt% is Fe, and 52 wt% is PO_4_. The Li content is 4.1 wt% and Al is present as impurity (0.3 wt%). 10.8 wt% of the black mass is taken up by graphite, O and others.

Lastly, an industrial BM is shown in Fig. 5i. Its pre-treatment differs from the one applied to earlier mentioned BMs; the battery cells are shredded entirely instead of disassembled manually (the latter including separation of anode and cathode). Also, the BM is pyrolyzed to remove organic components such as the binder and electrolyte components. Therefore, more impurities are to be expected. The BM consists of a multitude of compounds, such as NMC, LCO and LFP as well as graphite, which is used as anode active material and is not present in previous samples. Particles of Al and Cu foils are also present. These result from breakage of the cathode and anode collector foils, respectively. Al was present in some previous samples, whereas Cu was not. XRD-analysis confirms the presence of these phases, as well as Cu_0.2_MnNi_5.8_O_8_, Cu_0.85_Fe_0.1_O and Li_2_CO_3_. Also, a part of the Ni is present in metallic form. This is due to it being present in the metallic shell of the 18650 cells, which is not removed in industrial pre-treatment. According to ICP-OES-analysis, Co is the most prevalent element (18 wt%) in the industrial sample, followed by Ni (12 wt%), Mn (4.7 wt%) and Li (4.2 wt%). Al (2.8 wt%) and Cu (2.1 wt%) are both present in a small amount, as well as Fe (0.4 wt%). Other compounds and elements, such as graphite, binder, and O take up 55.4 wt%.

When comparing all these different BMs, it can be seen that there is a number of different cathode chemistries present in various electronic devices. Samples can be high in Co (LCO), Mn (LMO), Ni (NMC 1 and 2) or contain Fe and P (LFP). Sometimes, a blend of different chemistries is used (LMO + NMC, LCO + NMC 1 and 2). No direct link is found between the type of device and the cathode chemistry, suggesting that sorting per device will not reduce the variation in chemistries. Other elements such as Al are often present as impurities. It is important to note that large standard deviations of the Al content were found since in some batteries, the collector foil was so thin that it broke by the ultrasonic vibrations during the liberation process.

The industrial sample seems to be a mixture of all other investigated battery types, as Li, Co, Ni, Mn, Al, and Fe are all present. Mechanical processing and pyrolysis are generally able to remove most of the plastics originating from the module casings, glue and separating components as well as the pouch cell casings and separators^42^. The metallic Fe, which can originate from the cell casing of cylindrical cells, can be largely removed by magnetic separation^42^. In addition, it contains impurities of Cu and Al, likely from the current collectors. These are, on industrial scale, separated by multiple sieving steps and electrostatic separation, however, certain level of impurities is always present after mechanical processing^42^. Lastly, the industrial BM contains a lot of graphite, as seen on the SEM-image (Fig. 5i). This is the active material of the anode, which could be separated by flotation or removed by heat-treatment^42^. Alternatively, it can also be removed as residue after leaching^42^.

### Effect of black mass composition and H_2_O_2_ addition on leaching

A selection of BMs that were retrieved in the previous sections, as well as a pristine sample, were submitted to the same leaching conditions. This provides insight into the influence of BM composition on the leaching efficiency of the contained elements. The following conditions were chosen as a benchmark based on a literature survey^20,22,28,51^. All samples were submitted to a lixiviant with 2 M H_2_SO_4_ for 2 h at 50 °C and S/L of 60 g/L. The addition of H_2_O_2_ is varied from 0 to 4 vol% to study the required extent for a reductive agent, which improves dissolution of the TMs^52^. In order to perform leaching experiments on multiple BMs, only 0, 1 and 4 vol% of H_2_O_2_ addition were applied. The driving reaction in this leaching system is described in Eq. (2) ^22^. Note that this reaction assumes an equal presence of Ni, Co and Mn, which is not always the case in this study.

2 $$6 Li{Ni}_\frac{1}{3}{Mn}_\frac{1}{3}{Co}_\frac{1}{3}{O}_{2 s}+9{H}_{2}S{O}_{4 aq}+3 {H}_{2}{O}_{2 aq}\to 3 {Li}_{2}S{O}_{4 aq}+2 NiS{O}_{4 aq}+2 CoS{O}_{4 aq}+2 MnS{O}_{4 aq}+12 {H}_{2}O+3 {O}_{2 g}$$

**Figure 6:**
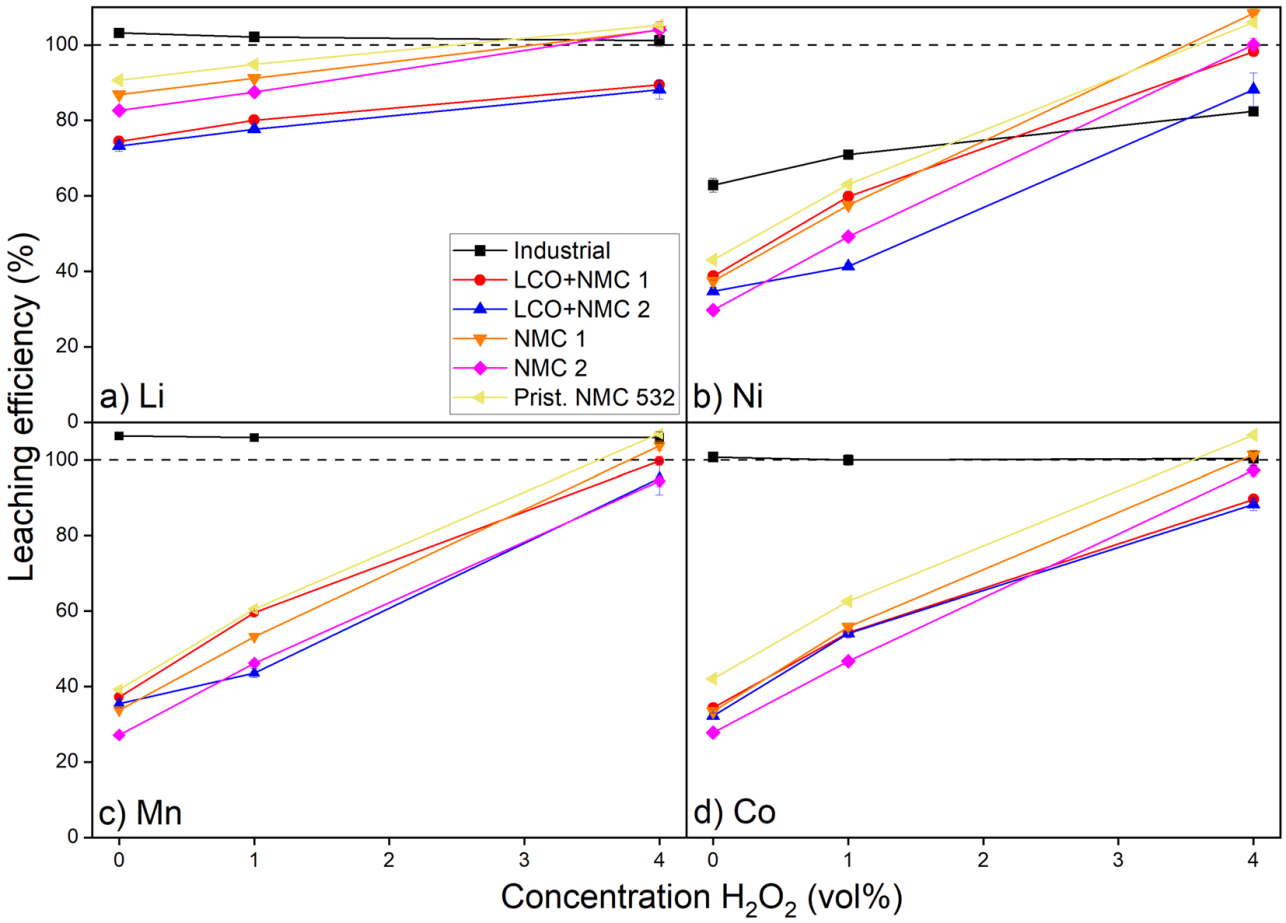
Leaching efficiencies of Li, Ni, Mn and Co from different BM samples as a function of the amount of H_2_O_2_ addition. Other leaching conditions are 2 M H_2_SO_4_, S/L = 60 g/L, T = 50 °C, t = 120 min.

The results of the leaching experiments for the different chemistries are illustrated in Fig. 6. Since the chosen manual liberation method resulted in a generally low amount of impurities (mostly Al), only the leaching efficiencies of the relevant elements; Li, Ni, Mn and Co are shown. It is important to note that sometimes the leaching efficiency exceeds 100%, which is not possible in practice. Evaporation of water from the PLS during the experimental phase could lead to an overestimation of elemental concentrations. Also, while both initial composition and leaching results were performed multiple times, the inherent heterogeneous nature of BM leads to an estimated error of 4–5%.

Without addition of H_2_O_2_, the leaching efficiency of all elements except Li is very poor. Li is leached between 68 and 90%, whereas dissolution of the TMs ranges between 25 and 45%. An increase in the H_2_O_2_ concentration generally improves the leaching efficiency of all relevant elements. This is already seen when 1 vol% of H_2_O_2_ solution is added, at which point the leaching efficiencies increase from 72 to 95% for Li and from 40 to 70% for the TMs. However, 100% leaching efficiency is not reached for all samples, despite their similar trends, even at 4 vol% H_2_O_2_. This is observed in the leaching of both mixed LCO and NMC chemistries, showing much lower efficiencies (approx. 88%, 89%, 93% and 97% for Li, Co, Ni and Mn, respectively), but comparable to each other. On the other hand, the two NMC samples (NMC 1 and NMC 2) show much more promising results, but with a slight discrepancy between the two samples (100% and 95% leaching efficiency, respectively) for TMs, while Li is leached for 100% in both cases. The pristine NMC 532 shows the best leaching behaviour, even at low (or zero) H_2_O_2_ concentration. At 4 vol% H_2_O_2_, both the TMs and Li are fully leached.

The observed trend concerning the H_2_O_2_ concentration, as well as the distinct leaching behaviour of Li compared to the TMs is in agreement with earlier studies^22,28^. It is to be expected that 100% leaching efficiency will be achieved for all BMs with further increase of the H_2_O_2_ concentrations^53^. Noticeably, however, the leaching trend of the industrial BM is completely different from all other BM samples. Strangely, Li, Co and Mn are fully leached without addition of any H_2_O_2_. However, at this point, Ni is only leached for 61% and does not exceed 80% at 4 vol% H_2_O_2_ solution. The high observed leaching efficiencies can be attributed to the low metal concentration in the feed, as seen in Table 1. It contains a lot of graphite, which does not dissolve during leaching but does contribute to the S/L, which was kept constant in all leaching experiments. Therefore, the total amount of metals in the feed is much lower relative to other samples. This dilution of the target metals by other components, such as graphite, plastics, binder and current collectors (Al, Cu) is also reported in other studies^54^. Also, it is known that pyrolysis of BMs containing graphite can induce reactions between the present metals and graphite, causing pre-reduction of the TMs^55^. As a consequence, leaching efficiencies are much higher at a lower addition of H_2_O_2_. Ni is the only element that does not fully dissolve, which is a result of it being present in metallic form, as observed earlier.

Li leaching generally shows a slightly different trend compared to the transition metals. At lower H_2_O_2_ concentrations it is not completely leached, but it varies from 70 to 90%. This is due to the good solubility of Li^+^ and the lower binding energy of Li and O, compared to the other TMs^28,56^. However, for Li to be fully leached, the solid particles need to be broken down first, enabling contact between the Li present inside these particles and the dissolving acid. On the other hand, the TMs dissolve most efficiently in oxidation state + II^31^. However, these are also present in state + III (Ni and Co) and + IV (Co and Mn)^57,58^. Hence, they need to be reduced before dissolution, which is manifested by the reaction with H_2_O_2_^53^. When the average oxidation state is higher, a larger amount of H_2_O_2_ is needed to reduce the TMs to the + II state^31^. The leaching results suggest a different magnitude of H_2_O_2_ consumption for the tested BMs, and therefore a different distribution of the oxidation numbers of the TMs in these samples. To study this in more detail, some of these samples have been subjected to leaching with an excess of H_2_O_2_ in combination with KMnO_4_-titration.

When leaching with 10 vol% H_2_O_2_ solution, all the Li and TMs in NMC 1, NMC 2, the industrial BM and the pristine NMC 532 were transferred to the PLS according to ICP-OES analysis. Afterwards, the remaining H_2_O_2_ was titrated with a KMnO_4_ solution to calculate the H_2_O_2_ consumption, which is expressed as moles of H_2_O_2_ consumed per mole of TMs. Results show that the dissolution of NMC 2 consumes more H_2_O_2_ compared to NMC 1 (0.91 and 0.87, respectively). Dissolution of the industrial BM requires about 3 times more H_2_O_2_ (2.7 mol per mole of TMs). The dissolution of the pristine NMC 532 results in the lowest ratio (0.67). These differences in consumption of the reducing agent indicate, according to Eq. (2), the difference in oxidation states of the TMs in the NMC samples. The fact that the industrial BM requires so much reducing agent compared to the other samples is unexpected. One explanation is the high content of impurities which can react with H_2_O_2_, such as Cu, Al and Fe^59–61^. Therefore, the high consumption is not only related to the oxidation number of the target TMs and cannot be directly compared with the other samples. The H_2_O_2_ consumption of NMC 2, NMC 1 and pristine (NMC 532) is in agreements with the leaching results observed earlier, as leaching efficiencies follows the same respective order NMC 2 > NMC 1 > pristine concluded for increasing oxidation states of TMs. The titration experiments suggest a higher average oxidation states of Ni and Co in the used samples (NMC 1 and 2) compared to pristine material, which directly influences their leaching efficiencies.

Generally, the leaching experiments show that it is possible to leach all the different chemistries together at high H_2_O_2_ concentration. It has to be noted that not all CAM chemistries available on the market were used in the leaching experiments. These may be present in an industrially processed BM, as is shown earlier, and lead to even more divergence in leaching efficiencies. To accommodate for variations in chemistries, a high amounts of reagents is needed, putting extra pressure on the sustainability of the recycling process as a whole. Also important to note is that in the leaching of the industrial BM, impurities end up in the PLS. Table S2 in the supplementary information shows the composition of the PLS, acquired after leaching the industrial BM with 4 vol% H_2_O_2_-solution. It can be seen that Cu, Al and Fe contaminate this PLS. In order for any recycled Li, Co, Ni and Mn products to meet industrial standards, these elements need to be removed. There are various methods to serve this purpose, such as solvent extraction and subsequent stripping and scrubbing steps, precipitation and recrystallization or electrowinning^62^. However, these steps can be energy intensive and produce (indirect) emissions.

## Conclusions

Li-ion batteries from a range of consumer electronics were systematically disassembled and characterized, highlighting possible sources of impurities that can contaminate the BM. Two main cell types were found: pouch- and 18650-cells. The main difference between them is the cell casing (consisting of plastic or steel, respectively). If the casing is excluded, the other components (separator, cathode and anode) have the same relative weight in both types of cells. The individual battery cells are the major component (> 71 wt%) of battery modules from consumer electronics. Internal wiring and glue/separating components take up a very small portion. The BMS takes up a larger relative weight in small modules and when fulfilling other needs besides safe (dis)charging. The casing is larger for modules with multiple cells due to a greater need of structural integrity. Major impurities after industrial BM processing are Al, Cu and graphite. However, the presence of Ni in metallic form, assumably originating from the metal casing on 18650 cells, was also observed. Next, the CAMs were liberated from those old battery cells and characterized. Results show that there is no link between the CAM chemistry and the type of application. Various chemical compositions of CAMs have been found in consumer LiBs, sometimes with multiple chemistries combined in single battery cells. As expected, industrial BM is a combination of many different cathode chemistries, as well as earlier mentioned impurities.

With the aim of exploring the possibilities for a universal hydrometallurgical process, we researched the influence of chemical composition on the leaching behaviour of Li, Ni, Mn and Co under the same H_2_SO_4_ leaching conditions (2 mol/L, 50 °C, 2 h, 400 rpm, 60 g BM/L) and a varying concentration of H_2_O_2_ (0–4 vol%). Although samples with different chemistries generally exhibited similar trends, there is a difference in the amount of H_2_O_2_ required for complete dissolution of all the TMs. The leaching efficiencies of all studied elements generally increased with additions of H_2_O_2_, except for the industrial BM. Pristine NMC 532 and one of the used NMC BMs showed complete dissolution in the presence of 4 vol% H_2_O_2_-solution, whereas other BMs reached between 80 or 90% efficiency under the same conditions. The industrial BM showed distinct behaviour. All metals except Ni were leached completely without the need for H_2_O_2_. The low Ni leaching efficiency was attributed to the presence of metallic nickel from the nickel-plated steel battery cell casing. The high leaching efficiencies of Li, Co and Mn in the industrial BM is attributed to their relatively low content compared to other samples (due to the high content of non-metallic impurities such as graphite). Lastly, variations in the oxidation states of Ni and Co from EoL batteries were found to affect the leaching process. This was confirmed by leaching with an excess of H_2_O_2_ followed by titration. This study shows that a universal hydrometallurgical leaching process can be designed to deal with the high variety of available chemistries in mixed BM coming from EoL Li-ion batteries in consumer waste. Nevertheless, this requires excess amount of additional reducing agents, such as H_2_O_2_, to achieve high leaching efficiencies before further purification and recovery.

### Supplementary Information

Supplementary Information.

## Data Availability

The datasets used and/or analysed during the current study are available at the 4TU Research data repository. 10.4121/fb1d44aa-8d83-44c5-ac6a-f18e6d43a42d.
