# Supplementary material for: A closer look at lithium-ion batteries in E-waste and the potential for a universal hydrometallurgical recycling process
Source: Sci Rep. 2024 Jul 19;14:16661. doi: 10.1038/s41598-024-67507-7 (PMC11271561; doi:10.1038/s41598-024-67507-7)
Supplement: Supplementary file 1 — Supplementary Information. [file 41598_2024_67507_MOESM1_ESM.docx]

Supplementary information: A Closer Look at Lithium-Ion Batteries in E-waste and the Potential for a Universal Hydrometallurgical Recycling Process

Johannes J.M.M. van de Ven ^1^, Yongxiang Yang ^1^ & Shoshan T. Abrahami ^1*^

^1^ Delft University of Technology, Department of Materials Science and Engineering, Mekelweg 2, 2628 CD Delft, The Netherlands

# Experimental


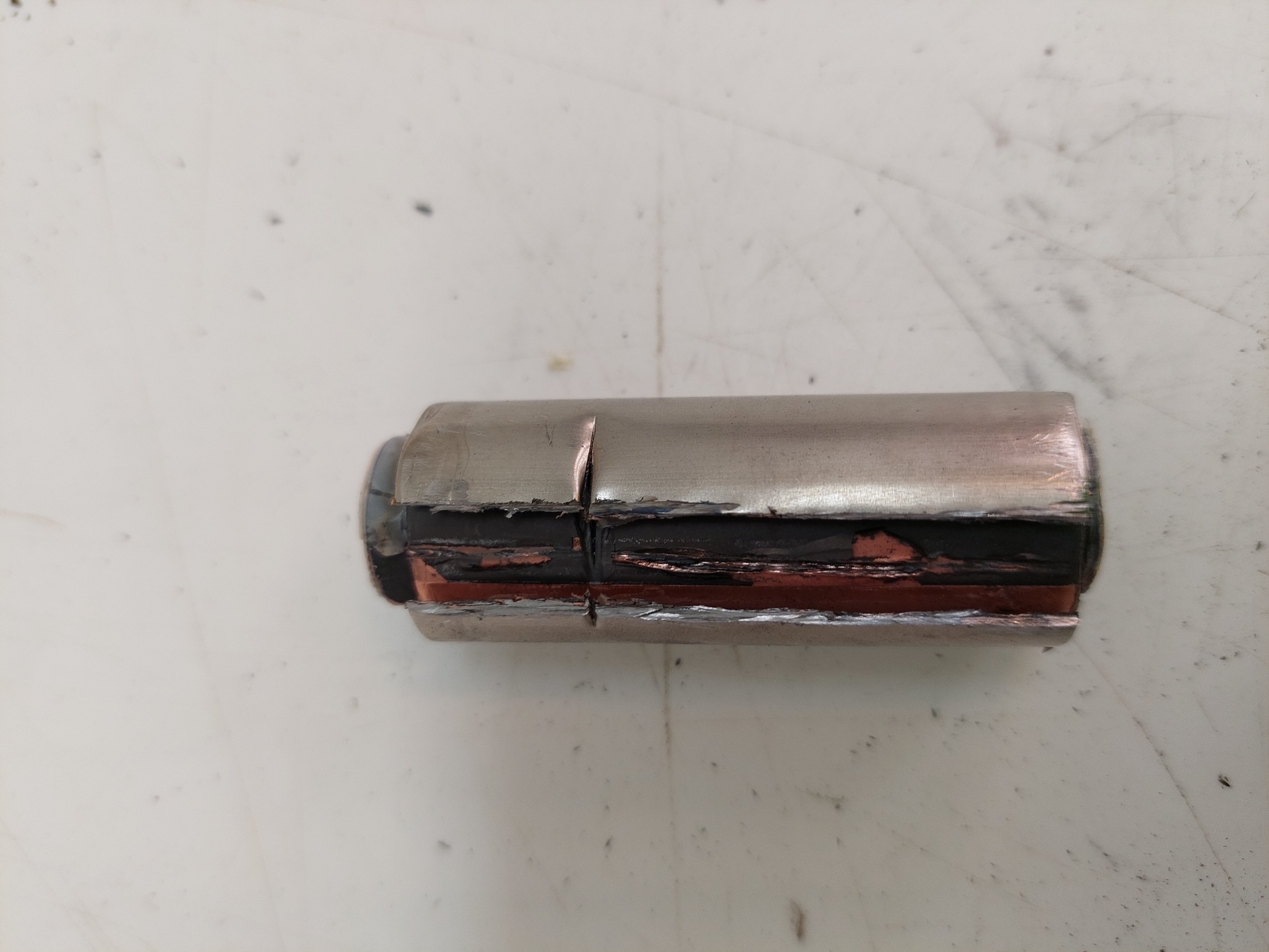


Figure S1: 18650-type cell after removal of top and bottom and making a longitudinal cut.

# XRD-SEM results

Table S1: Explanation of the sub images in Fig.5. In addition, the XRD results of the corresponding depicted BMs are shown.

| Image | Black mass name | Compound | Grain size | XRD results |
| --- | --- | --- | --- | --- |
| a) | NMC 1 | NMC | 1 – 4 µm Individual  8 – 20 µm Coagulated | LiNi_x_Mn_y_Co_z_O_2_ |
| b) | NMC 2 | NMC | 1 – 4 µm Individual  8 – 20 µm Coagulated | LiNi_x_Mn_y_Co_z_O_2_ |
| c) | LCO 1 | LCO | 2 – 40 µm | LiCoO_2_ + CoO_2_ |
| d) | LCO 2 | LCO | 10 – 30 µm | LiCoO_2_ |
| e) | LMO + NMC | NMC  LMO | 1 – 4 µm Individual  8 – 20 µm Coagulated  7 – 40 µm | LiMn_2_O_4_ + Li_1.2_Mn_0.6_Ni_0.2_O_2_ |
| f) | LCO + NMC 1 | NMC  LCO | 1 – 4 µm Individual  8 – 20 µm Coagulated  10 – 30 µm | LiCoO_2_ + LiNi_x_Mn_y_Co_z_O_2_ |
| g) | LCO + NMC 2 | NMC  LCO | 1 – 4 µm  3 – 30 µm | LiCoO_2_ + LiNi_x_Mn_y_Co_z_O_2_ |
| h) | LFP | LFP | 1 – 15 µm | LiFePO_4_ + FePO_4_ + C |
| i) | Industrial | C  NMC  LCO  LFP  Al, Cu | 10 – 25 µm  10 – 50 µm  10 – 20 µm  4 – 8 µm  Variable | C + Ni + CoO + Cu_0.2_MnNi_5.8_O_8_ + LiMn_0.8_Ni_1.2_O_4_ + Cu_0.85_Fe_0.1_O + Li_2_CO_3_ + Li_3_PO_4_ |

XRD suggests an equal molar distribution of the TMs in NMC 1 and NMC 2, as well as the mixed oxide in LCO + NMC 1 and LCO + NMC 2. This contradicts the XRF measurements. However, distinguishing NMC cathode powers with different ratios of TMs (111, 532, 811 etc.) by XRD-analysis is very difficult due to their nearly identical diffractograms ^[1]^. Therefore, only the ICP-OES-results are considered for distinguishing the exact cathode chemistry.

# ICP-OES results for industrial BM leaching

Table S2: Elemental concentrations (g/L) of Li, Co, Ni, Mn, Al, Fe and Cu in the PLS after leaching industrial BM. Leaching conditions were 2 mol/L H_2_SO_4_, 4 vol% H_2_O_2_-solution, S/L = 60 g/L, T = 50 °C and t = 120 min.

| Element | Li | Co | Ni | Mn | Al | Fe | Cu |
| --- | --- | --- | --- | --- | --- | --- | --- |
| Concentration (g/L) | 2.6 | 10.8 | 5.9 | 3.0 | 1.8 | 0.36 | 0.86 |

# References

1. Azhari, L. *et al.* Effects of Extended Aqueous Processing on Structure, Chemistry, and Performance of Polycrystalline LiNi_x_Mn_y_Co_z_O_2_Cathode Powders. *ACS Appl Mater Interfaces* **12**, 57963–57974 (2020).
